# Disadvantages (?) of Menthol

**Published:** 1911-08

**Authors:** 


### Disadvantages (?) of Menthol

H. Triboulet, *La Clinique,* January 13, 1911, mentions irritation of various kinds, due to the use of menthol in coryza; acute conjunctivitis due to a proprietary powder; and a dermatitis of the face, resembling erysipelas, due to a hay fever snuff, containing menthol. He naively states that he has never himself encountered nasal, pharyngeal and laryngeal irritation, having never used a solution stronger than 10 per cent.

It would seem that such disadvantages are due to a misapprehension of the action and, more particularly of the concentration of menthol to be used. Aside from a slight antiseptic action, and the sense of comfort which menthol affords in weak solutions, the value of this agent—or of oil of peppermint—is due to its stimulation of local blood supply which naturally enough, would bring about the disadvantages claimed if too strong solutions are used.

Long ago, we used a 10 per cent. solution of menthol in purpetrol for erysipelas and local sepsis and with very prompt results. We occasionally employ a 10 per cent., more often a 5 per cent. solution in purpetrol as an endo-gastric spray, the actual dosage by this method being very small. For ordinary use on mucous membranes, 2 per cent. should rarely be exceeded and 1 or even ½ per cent. is usually more soothing. It ought to be obvious that menthol is not adapted to use on the conjunctiva, or in the urethra and we have even been very cautious as to employing it about the anus although a weak oily solution in the lower bowel or even applied by pledgets of cotton to the anus itself, may act favorably.

In this connection, we may call attention to the fact that peppermint is by no means an inefficient remedy nor one that can be used with impunity. We have seen syncope and transient deafness caused by the taking of a teaspoonful of the essential oil, but with recovery.

